# Organic tracers in fine and coarse aerosols at an urban Mediterranean site: contribution of biomass burning and biogenic emissions

**DOI:** 10.1007/s11356-024-32789-x

**Published:** 2024-03-11

**Authors:** Álvaro Clemente, Eduardo Yubero, Jose F. Nicolás, Javier Crespo, Nuria Galindo

**Affiliations:** Atmospheric Pollution Laboratory (LCA), Department of Applied Physics, Miguel Hernández University, Avenida de la Universidad S/N, 03202 Elche, Spain

**Keywords:** Carbohydrates, PM_1_, PM_10_, Size distributions, Biomass burning

## Abstract

The concentrations of anhydrosugars (levoglucosan, mannosan, and galactosan), polyols (inositol, xylitol, sorbitol, and mannitol), and glucose were measured in PM_1_ and PM_10_ samples collected during 1 year at a traffic site in the city of Elche (southeastern Spain). Levoglucosan, mannosan, and galactosan were mainly found in the PM_1_ fraction since they are mainly emitted from biomass burning (BB). Likewise, inositol, xylitol, and sorbitol were primarily distributed in the fine mode, suggesting a non-negligible contribution from anthropogenic sources (specifically BB) to the levels of these compounds. This was supported by their seasonal variations, with higher concentrations during winter, and their correlations with levoglucosan concentrations. The average contributions of biomass burning and biogenic sources to OC and PM levels were calculated using levoglucosan and mannitol, respectively, as tracers. On average, BB accounted for 12% and 16% of the OC in PM_1_ and PM_10_, while the estimated contribution of fungal spores to OC and PM_10_ levels was 1.2 and 0.8%, respectively. The results of the present study suggest that, at least in the study area, most sugar alcohols are not appropriate tracers of biogenic emissions.

## Introduction

Characterizing the chemical composition of atmospheric aerosols is essential to identify pollution sources, evaluate their effects on human health and ecosystems, and associate aerosol optical properties with visibility impairment and climate change (Chow et al. 2015). Aerosols consist of organic and inorganic species. Organic matter is a major constituent of atmospheric particulate matter (PM), accounting for 20–90% of submicron particles (Jiménez et al. 2009). The organic fraction of atmospheric aerosols includes primary and secondary organic components. Primary organic aerosols are directly emitted by different sources including fossil fuel combustion, biomass burning, and biogenic emissions, while secondary organic aerosols are formed by atmospheric oxidation of gaseous precursors of both biogenic and anthropogenic origin (Jiménez et al. 2009; Samaké et al. 2020).

Organic aerosols contain chemical species that can be used as tracers of specific pollution sources. Among these species, sugar compounds (also named carbohydrates or saccharides) have received increased attention in recent years (Gonçalves et al. 2021; Samaké et al. 2020; Theodosi et al. 2018; Zhu et al. 2022) since they are reliable markers of different biogenic and anthropogenic sources and can provide information on the transport routes of atmospheric particles (Fraser and Lakshmanan 2000; Marynowski and Simoneit 2022; Rathnayake et al. 2016; Vincenti et al. 2022). Anhydrosaccharides (specifically levoglucosan and its isomers mannosan and galactosan) are pyrolytic degradation products of cellulose and hemicellulose (Marynowski and Simoneit 2022; Vicente and Alves 2018; Vincenti et al. 2022) and have been widely used as tracers of biomass burning (de Oliveira Alves et al. 2015; Galindo et al. 2021; Monteiro et al. 2018; Zhu et al. 2022). Sugar alcohols mainly come from fungal spores (e.g., mannitol and arabitol; Bauer et al. 2008) and soil dust (e.g., sorbitol and xylitol; Simoneit et al. 2004), while primary saccharides, such as glucose and sucrose, can be used as tracers of plant materials (e.g., pollen and plant fragments) and emissions from soils (Fu et al. 2012; Simoneit et al. 2004; Vincenti et al. 2022; Zhu et al. 2022). Although it has been reported that these last two classes of carbohydrates can also be emitted from biomass burning (Simoneit et al. 2004; Vincenti et al. 2022; Yttri et al. 2007), they have been used to estimate the influence of primary biogenic emissions (i.e., particles released into the atmosphere from the biosphere, including pollen and plant debris, fungal spores, or bacteria) to the levels of organic carbon and PM in a number of previous works (Cao et al. 2022; Casotto et al. 2023; Gonçalves et al. 2021; Jia et al. 2010; Rathnayake et al. 2016; Samaké et al. 2019a; Xu et al. 2020). Despite all this, there are still few studies focusing on the sugar content of atmospheric aerosols in the Mediterranean basin. In a work performed at a remote site in Crete, Theodosi et al. (2018) analyzed the carbohydrate content of PM_10_ samples collected over a 2-year period and estimated an annual average contribution from biomass burning to organic carbon of 13%. Samaké et al. (2019a,b) used the concentrations of different polyols and glucose to evaluate the influence of primary biogenic emissions on the levels of organic matter in PM_10_ at urban and rural sites in France. In Spain, anhydrosugars and glucose, together with other organic markers, were simultaneously measured in Granada and Barcelona (van Droogue et al. 2022) in order to assess the importance of primary and secondary organic aerosols under different meteorological conditions. A wide range of sugar compounds were analyzed in the ambient air of León (northwest of Spain) during 1 year with the aim of studying the relationship between their concentrations and meteorological factors, mainly rainfall.

In all of the mentioned studies, carbohydrate concentrations were only measured in PM_10_ samples. In fact, to the best of our knowledge, this is the first time that the sugar content was simultaneously analyzed in two size fractions (PM_1_ and PM_10_) in the western Mediterranean. The knowledge of the size distribution of these molecular markers can provide further insights into their sources. The main purpose of the present work is to determine the concentration of anhydrosugars, sugars, and sugar alcohols in PM_1_ and PM_10_ samples in order to evaluate the influence of biomass burning and biogenic sources at a typical Mediterranean urban site.

## Materials and methods

### Sample collection

Twenty-four-hour PM_1_ and PM_10_ samples were collected three times a week using two Derenda 3.1 low-volume samplers (2.3 m^3^ h^−1^) according to the EN 12341 European Norm (CEN/TC 264 2023). Samplers were installed on the first floor of a seven-story building occupied by the local Environmental Office. The measurement site was located in the city center of Elche (Spain), on a street having two lanes on the same direction. One of the lanes was converted into a bus lane by mid-2021, leaving only one lane for general traffic. A detailed description of the characteristics of the study area can be found in Nicolás et al. (2020).

Around 160 samples of each fraction were collected onto quartz fiber filters between November 2020 and November 2021. Mass concentrations were determined gravimetrically using an Ohaus AP250D analytical balance. Filters were kept in controlled conditions (20 ± 1 °C and 50 ± 5% relative humidity) for at least 24 h before weighting.

### Chemical analyses

A quarter of each filter was extracted in 3.5 ml of ultrapure water by ultrasonic bath agitation for 45 min. The extracts were then filtered through 0.45 µm syringe filters (13 mm) to remove insoluble materials before analysis. Sugar anhydrides (levoglucosan, mannosan, and galactosan), sugar alcohols (inositol, xylitol, sorbitol, and mannitol), and glucose were quantified by high-performance anion exchange chromatography with pulsed amperometric detection (HPAEC-PAD). A Thermo Scientific Dionex Integrion system equipped with a two-way valve was used. This setting allows to alternately run two NaOH solutions with different concentrations. The analytical column was a 250 × 4 mm Dionex Carbopac PA10, and runs were initiated with 25 mM NaOH for 25 min at a flow rate of 0.5 ml min^−1^. Then, the column was cleaned with 200 mM NaOH for 8 min and re-equilibrated with 25 mM NaOH for 17 min before the injection of the next sample. For the amperometric detection, a gold working electrode was used.

The content of organic carbon (OC) and elemental carbon (EC) of the samples was quantified using the thermal-optical transmittance instrument from Sunset Laboratory. A rectangular filter punch of 1.5 cm^2^ was cut from each filter and analyzed using the EUSAAR2 protocol (Cavalli and Putaud 2010).

Field (*n* = 12) and laboratory blanks (*n* = 24) were also analyzed by the different techniques, and the concentrations were subtracted from the values obtained for each sample. The limits of detection (LOD) of sugar compounds were determined as the minimum concentration that was visible in the chromatogram and produced a peak height at least three times the signal-to-noise ratio. LODs ranged from 0.002 to 0.04 µg ml^−1^.

### Gaseous pollutant concentrations and meteorological variables

Daily concentrations of gaseous pollutants and meteorological data were obtained from two stations of the Air Quality Regional Network located ~ 2 km and 3.5 km, respectively, from the sampling site. During the measurement period, the average temperature was 19.5 °C, ranging from 13.6 °C in winter to 26.3 °C in summer. The accumulated precipitation during the sampling days was only 118 mm and was primarily concentrated in spring (~100 mm).

## Results and discussion

### Average concentrations and size distribution of saccharides

Mean concentrations of PM, carbonaceous species, and carbohydrates during the sampling period are shown in Table 1. Galactosan, mannosan, xylitol, and sorbitol were not detected in a significant number of samples (between 18 and 48%). For this reason, values below detection limits were replaced by half of the minimum detected value to calculate the annual mean concentration.

**Table 1:** Mean concentrations (±standard deviations) of PM, OC, EC, and carbohydrates averaged for the whole study period. PM, OC, and EC levels are expressed in µg m^−3^, while carbohydrate concentrations are given in ng m^−3^

	Mean ± SD		Max		Min	
	PM_1_	PM_10_	PM_1_	PM_10_	PM_1_	PM_10_
PM	9.3 ± 4.7	27.9 ± 17.0	39.4	149.2	3.4	7.4
OC	3.6 ± 1.0	4.9 ± 1.4	7.4	10.2	2.0	2.7
EC	0.7 ± 0.4	1.1 ± 0.4	2.1	2.6	0.2	0.3
Levoglucosan	20.6 ± 23.4	34.1 ± 31.0	121.8	166.5	0.7	1.8
Mannosan	3.2 ± 4.8	4.6 ± 6.4	19.5	30.8	< LD	< LD
Galactosan	1.3 ± 2.1	2.1 ± 3.4	10.1	18.4	< LD	< LD
Inositol	3.3 ± 1.8	4.4 ± 2.2	11.1	13.2	< LD	0.3
Xylitol	0.6 ± 0.6	0.8 ± 0.7	2.5	3.5	< LD	< LD
Sorbitol	0.8 ± 0.6	1.1 ± 0.7	2.5	4.5	< LD	< LD
Mannitol	1.5 ± 0.7	4.5 ± 2.7	4.2	15.9	< LD	< LD
Glucose	6.4 ± 3.4	20.0 ± 10.7	18.2	60.9	1.7	5.4

< *LD*, minimum concentrations below detection limits

The carbonaceous fraction (OC + EC) accounted for 22% and 47%, respectively, of the PM_10_ and PM_1_ average mass concentrations, in agreement with the results previously observed at the same sampling site (Clemente et al. 2022). The value for PM_10_ was in the range of those reported for other urban and suburban areas, which generally vary between 20 and 35% depending on the season of the year and the specific characteristics of the sampling site (Di Vaio et al. 2016; Kılavuz et al. 2019; Megido et al. 2016; Waked et al. 2014). As expected, the contribution of carbonaceous species to PM_1_ levels was notably higher than that of PM_10_, since a significant portion of the coarse fraction is made up of mineral dust and sea salt, which are minor components of submicron particles (Ariola et al. 2006; Galindo et al. 2020; Titos et al. 2014).

Biomass burning (BB) tracers (levoglucosan, mannosan, and galactosan) generally showed lower concentrations than those reported in a number of previous works (Table 2). For instance, in the present study, the mean levoglucosan concentration in the PM_10_ fraction was 34 ng m^−3^, varying between 14 ng m^−3^ in summer (June, July, and August) and 68 ng m^−3^ in winter (December, January, and February), while at other urban sites in Europe, levoglucosan levels were commonly much higher, particularly during winter. This indicates lower PM emissions from residential wood burning in the city of Elche, which is partly due to its mild winter temperatures compared to more northern European regions. Surprisingly, the average annual levoglucosan concentration measured in the present study was higher than that obtained in the city of León (Oduber et al. 2021), located in the northwest of Spain, despite winters being much colder and longer in León (5 ± 3 °C) than in Elche.

**Table 2:** Concentrations of saccharides (in ng m^−3^) reported in the literature at urban sites in Europe

PM	Site	Levoglucosan	Mannosan	Galactosan	Reference
PM_1_	Elche (Spain)	20.6	3.2	1.3	This study
PM_10_	Elche (Spain)	34.1	4.6	2.1	This study
PM_10_	Coimbra (Portugal)	40–444^a^	6.1–81.8^a^	2.5–28.6^a^	Gonçalves et al. 2021
PM_10_	Budapest (Hungary)	387	28	16	Salma et al. 2017
PM_10_	Leicester (UK)	45 (20–72^a^)			Cordell et al. 2016
PM_10_	Antwerpen (Belgium)	61 (18–185^a^)	6.9 (1.6–20^a^)	2.6 (0.8–7.4^a^)	Maenhaut et al. 2016
PM_10_	Rome (Italy)	277^b^			Perrino et al. 2019
PM_10_	Central London (UK)	160^c^	22^c^	10^c^	Fuller et al. 2014
PM_10_	Sosnowiec (Poland)	123–1009^a^	17.9–174^a^	1.8–27.6^a^	Marynowski et al. 2020
PM_10_	León (Spain)	17	10	5	Oduber et al. 2021
PM_10_	Barcelona (Spain)	119.8^e^	11.4^e^	13.6^e^	van Drooge et al. 2022
PM_10_	Granada (Spain)	325.8^e^	23.7^e^	36.1^e^	van Drooge et al. 2022
TSP^d^	Belgrade (Serbia)	425^e^	56^f^	25^e^	Zangrando et al. 2016
PM_1_	Brno (Czech Republic)	27–220^a^	7.7–41^a^	3.5–11.8^a^	Křůmal et al. 2010

^a^Summer and winter averages, respectively. ^b^Average concentration during the heating season (November–March). ^c^Average values between January and February. ^d^Total suspended particles. ^e^Winter averages. ^f^Average values between September and December

The concentrations of sugar alcohols measured in Elche were in general much lower than the values found at other urban sites worldwide, although concentrations show substantial site-to-site differences. For example, Gonçalves et al. (2021) reported concentrations of xylitol in PM_10_ ranging from 3 ng m^−3^ in summer to 20 ng m^−3^ in winter at an urban background site in Coimbra (Portugal), while mannitol concentrations were around 20 ng m^−3^ during both summer and winter. In a study performed at different urban sites in Iowa, Rathnayake et al. (2016) also found mannitol levels remarkably higher than the ones registered in Elche (32 ng m^−3^). In contrast, average concentrations measured in PM_2.5_ at an urban site in Brno (Czech Republic) were between 1.8 and 4.3 ng m^−3^ for inositol, 1.0 and 4.2 ng m^−3^ for sorbitol, and 1.6 and 7.4 ng m^−3^ for mannitol, depending of the season of the year (Mikuška et al. 2017). Similarly, the levels of different polyols recently reported by Cao et al. (2022) and Kang et al. (2018) in Nanjing and Beijing (China), respectively, for the PM_2.5_ fraction were more comparable to the ones found in the present study for PM_1_.

The average concentration of glucose in PM_10_ (20 ng m^−3^) was in the lower range of those measured at other urban sites. The mean concentration of glucose in TSP registered in Belgrade was 22 ng m^−3^ (Zangrando et al. 2016), while higher concentrations were reported in PM_10_ in Iowa (32 ng m^−3^; Rathnayake et al. 2016), the Polish city of Sosnowiec (between 63 ng m^−3^ in winter and 291 ng m^−3^ in spring; Marynowski et al. 2020) and Beijing (46 ng m^−3^; Liang et al. 2016).

Since sugars and sugar alcohols are commonly used as tracers of primary biogenic aerosols containing pollen, plant debris, and fungal spores, the relatively low levels measured in this study suggest a limited contribution from biogenic sources to organic aerosols at our sampling site, most likely due to the lack of vegetation cover in nearby areas. Vegetation coverage has been found to affect the levels of glucose and sugar alcohols such as mannitol, as reported by Rathnayake et al. (2016), who found higher glucose and mannitol concentrations in forested rural areas of the Midwestern United States than at urban sites. The lower emissions from wood burning could also be a reason for the lower levels of some polyols observed in this study compared to other urban locations, since BB has been identified as a possible source of these compounds, as commented in the Introduction.

**Fig. 1:**
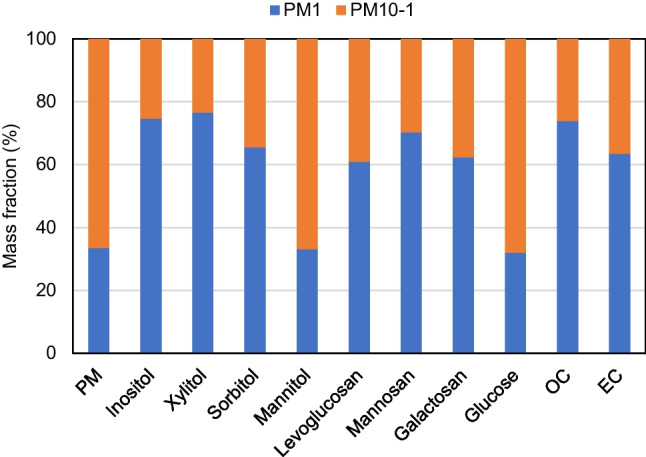
Average relative distribution of PM and carbonaceous components between the submicron and coarse fractions

Figure 1 shows the distribution of PM and the measured chemical components between PM_1_ and PM_10-1_. Approximately two-thirds of PM was distributed in the supermicron fraction, in line with other studies performed in the Mediterranean basin (Pikridas et al. 2018; Titos et al. 2014). This is due to a significant contribution from road dust resuspension and Saharan mineral dust to the PM_10-1_ fraction (Nicolás et al. 2009; Titos et al. 2014). OC and EC were mainly found in the submicron fraction, which was not unexpected since at the sampling site they are mainly associated to primary emissions from fossil fuel combustion and, in the case of OC, to secondary organic aerosol formation, and both processes mostly generate fine-mode particles (Jaffrezo et al. 2005).

On average, more than 60% of monosaccharide anhydrides were associated with submicron particles due to their origin from BB emissions (Blumberger et al. 2019). The presence of these compounds in particles larger than 1 μm could be associated to crop waste burning, which occurs at lower temperatures than wood burning and tends to generate coarser particles (Blumberger et al. 2019). This might be the reason why only around 40% of levoglucosan was distributed in the submicron fraction during summer when emissions from wood burning are expected to be very low.

Polyols, except mannitol, were mainly distributed in the fine fraction, which suggests that BB could be a likely source of these compounds, as already proposed by other studies (Liang et al. 2016; Yttri et al. 2007). In contrast, the predominant association of mannitol with the coarse fraction points to primary biogenic emissions (fungal spores) as a major source of this polyol (Carvalho et al. 2003).

Almost 70% of glucose was associated with particles larger than 1 μm, most likely because it mainly comes from plant fragments and soils. The size distributions of glucose have been examined in some previous works with contrasting results. In a recent work performed in Beijing (China) (Xu et al. 2020), glucose was also present primarily in the coarse fraction. Similar results were reported by Samaké et al. (2019b) in France and by Carvalho et al. (2003) in a Finnish forest. However, glucose was found to be more abundant in fine particles at a suburban site in Elverum (Norway) during winter (Yttri et al. 2007) and at the research station Melpitz, located in an agricultural area in Germany (Carvalho et al. 2003). Glucose in fine particles has been associated to its presence in fragmented pollen grains (Yttri et al. 2007).

### Seasonal variability of saccharide compounds

Table 3 presents seasonal concentrations of the analyzed carbohydrates in both PM_1_ and PM_10_. The levels of anhydrosugars were maxima in winter and minima during summer, as widely reported in the literature (Cao et al. 2022; Fu et al. 2012; Gonçalves et al. 2021; Jia et al. 2010; Křůmal et al. 2010; Marynowski et al. 2020). The use of wood burning for domestic heating only occurs to a significant extent during the cold season and progressively decreases with increasing temperature, as suggested by the negative correlations between levoglucosan, mannosan, and galactosan, and ambient temperature (*r* between 0.70 and 0.76, following a power law). Additionally, agricultural waste burning in the study area is not generally allowed between early June and mid-October. Similar results from the correlation analysis between levoglucosan concentrations and ambient temperature were obtained by Perrino et al. (2019) in Rome. On the other hand, anhydrosaccharides showed moderate negative correlations with ozone concentrations (*r* between 0.55 and 0.64). The oxidation with OH radicals, formed from ozone photolysis, has been proposed as the dominant removal mechanism for levoglucosan and its isomers (Bhattarai et al. 2019). Therefore, these results suggest that the degradation of anhydrosaccharides during summer under strong oxidation conditions could contribute to the lower concentrations during this season, as pointed out in previous studies (Xiao et al. 2018).

**Table 3:** Average concentrations of saccharides for each season (ng m^−3^)

	Winter		Spring		Summer		Autumn	
	PM_1_	PM_10_	PM_1_	PM_10_	PM_1_	PM_10_	PM_1_	PM_10_
Levoglucosan	45.4	67.5	13.2	30.3	4.8	12.9	19.5	28.6
Mannosan	8.0	11.9	2.2	4.2	*	*	1.3	2.8
Galactosan	3.4	6.0	0.8	1.7	*	*	*	1.3
Inositol	4.0	5.2	4.0	5.8	2.1	3.2	2.5	3.3
Xylitol	1.2	1.6	0.4	0.6	0.4	0.4	*	1.2
Sorbitol	1.2	1.6	0.5	0.9	0.3	0.7	1.3	1.5
Mannitol	1.9	3.6	1.3	4.9	1.3	5.0	1.5	4.4
Glucose	4.9	14.9	8.4	25.7	6.1	16.6	6.1	20.8

Winter: December, January, and February; Spring: March, April, and May; Summer: June, July, and August; Autumn: September, October, and November. *Values not shown because these components were not detected in most of the samples collected during summer and/or autumn

Glucose concentrations in both PM fractions were highest from March to early June as a result of the spring blooming season, in agreement with the findings of previous studies (Fu et al. 2012; Oduber et al. 2021; Xu et al. 2020).

With the exception of mannitol in the PM_10_ fraction, the concentrations of polyols at the sampling site were maxima during winter. This outcome could be attributed to higher emission rates of these compounds from biomass burning during the cold months. To explore this further, correlations between the concentrations of polyols and levoglucosan were performed. The results from this analysis (Table 4) suggest that biomass combustion can indeed be considered as a non-negligible source of polyols in the fine mode, particularly for xylitol. This observation is in line with the outcomes of Gonçalves et al. (2021) who also found strong correlations between xylitol and levoglucosan at roadside and urban background sites in Coimbra (Portugal).

**Table 4:** Pearson correlation coefficients between polyols and levoglucosan

	PM_1_	PM_10_
	Levoglucosan	
Inositol	0.54*	0.49*
Xylitol	0.84*	0.77*
Sorbitol	0.55*	0.52*
Mannitol	0.50*	0.00

^*^Correlations were statistically significant (*p* < 0.05)

Mannitol in PM_10_ did not correlate with levoglucosan, which indicates that biomass burning is not a significant source of this compound, at least in the coarse fraction. In fact, higher concentrations of PM_10_-bound mannitol were found during summer and spring, suggesting that biogenic emissions are the most important source of this compound. The positive correlation between mannitol and glucose in PM_10_ (*r* = 0.46, *p* < 0.05) indicates that both components share some common origin. Previous studies have also found a relationship between mannitol and glucose. For instance, Samaké et al. (2019b) observed the same seasonal cycle for both compounds, with maximum concentrations in spring, pointing to elevated biogenic emissions during this season due to higher biological metabolic activities. Alternatively, Oduber et al. (2021) found a significant correlation between mannitol and pollen concentrations, probably because this polyol is also present in different families of plants (Burshtein et al. 2011).

### Estimations of the contribution of biomass burning and biogenic emissions

Emission ratios can be used to estimate the contribution of different sources to PM levels (Li et al. 2021; Shahid et al. 2019; Titos et al. 2017). This approach assumes a fixed emission ratio between a compound emitted from a certain source and a tracer from that source (Li et al. 2021). Although the macro-tracer method has been extensively applied to estimate the contribution of BB to OC and PM concentrations (e.g., Galindo et al. 2021; Kirchsteiger et al. 2020; Stracquadanio et al. 2019; Theodosi et al. 2018), it is important to bear in mind that tracer emissions depend on multiple factors such as the type of biomass burned, moisture content, and combustion conditions, leading to uncertainty in the quantification of the real contribution of BB to aerosol levels. The protocol used to determine OC concentrations also adds uncertainty to estimates of the BB contribution since emission ratios are based on OC measurements.

**Fig. 2:**
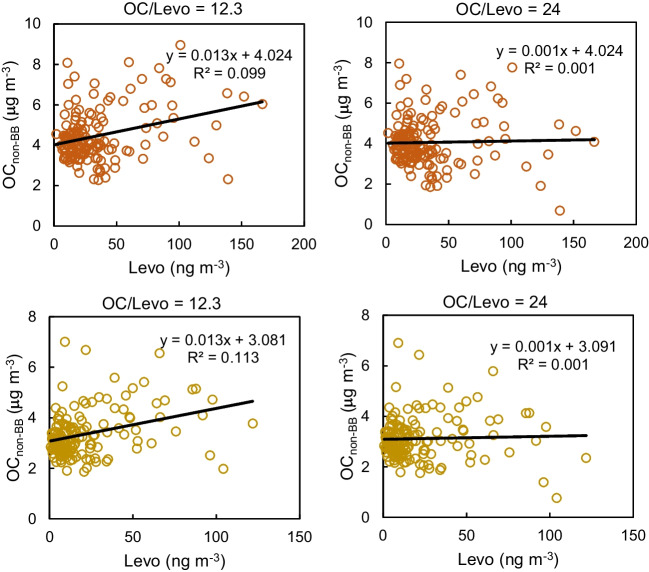
Correlation between non-biomass burning OC (OC_non-BB_) and levoglucosan concentrations. OC_non-BB_ levels were estimated from two OC/levoglucosan ratios for PM_10_ (top) and PM_1_ (bottom)

In the present work, OC/levoglucosan ratios at the sampling site were optimized using the method described by Nirmalkar et al. (2020) in order to estimate the contribution of biomass burning to OC concentrations in both PM_1_ and PM_10_. The first step of this approach consists of calculating the fraction of OC due to biomass burning emissions (OC_BB_) by multiplying levoglucosan concentrations by a given OC/levoglucosan ratio. Second, the proportion of OC non-attributable to biomass burning (OC_non-BB_) was estimated by subtracting OC_BB_ from the total OC. Finally, OC_non-BB_ was correlated against levoglucosan. If the estimated OC_non-bb_ does not contain OC from biomass burning, both the regression slope and *R*^2^ will be close to zero. As a starting point for this calculation, we used the ratio of 12.3 obtained for a residential area close to the sampling site (Galindo et al. 2021). Then, the value of this ratio was progressively increased until both the regression slope and *R*^2^ were close to zero. The optimized OC/levoglucosan ratio for our measurement site was 24 for both PM_1_ and PM_10_ (Fig. 2). This ratio was considerably higher than that estimated for PM_10_ at the residential site located in the surroundings of the city of Elche (Galindo et al. 2021). This could be explained considering that biomass combustion mainly occurs outside the city, and emissions from this source are then transported to the city center. During transport, the loss of levoglucosan by photochemical aging (Bhattarai et al. 2019; Vicente and Alves 2018) may lead to an increase in the OC/levoglucosan ratio.

The concentrations of OC from biomass burning in both PM_1_ and PM_10_ estimated using a conversion factor of 24 are presented in Table 5. The ratios between levoglucosan and its isomers, commonly used to identify the type of wood burnt (Janoszka and Czaplicka 2022; Shahid et al. 2019), are also shown. Higher levoglucosan/mannosan and levoglucosan/galactosan ratios have been reported for hard wood burning than for soft wood burning (Fine et al. 2004; Schmidl et al. 2011; Vicente and Alves 2018). These ratios were only calculated for the winter period, when biomass burning accounts for the highest percentage of measured concentrations.

**Table 5:** Contribution of biomass burning (BB) to OC levels. Average levoglucosan to mannosan and levoglucosan to galactosan ratios are also shown

	OC_BB_ PM_1_		OC_BB_ PM_10_			
	µg m^−3^	% of OC	µg m^−3^	% of OC	Levo/Man	Levo/Gal
Whole period	0.49 ± 0.56	12 ± 11	0.82 ± 0.75	16 ± 11	–	–
Winter	1.11 ± 0.76	24 ± 16	1.62 ± 1.04	26 ± 16	6.2	15.1

Winter: December, January, and February

**Fig. 3:**
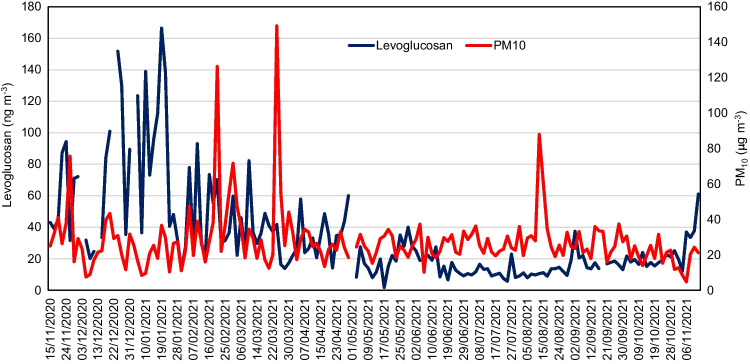
Variability of daily concentrations of levoglucosan and PM_10_ in Elche during the study period

The average contribution of BB to OC was comparable to that calculated for the same site using soluble potassium as a tracer for this source (15% three-year average; Galindo et al. 2019). However, it was somewhat lower than that found at the residential area close to the urban sampling site (30% during winter; Galindo et al. 2021), where wood combustion for house heating is frequent on cold days. The values obtained in the present study were lower than those reported for other urban areas in Europe, even those located in southern European countries. For instance, Benetello et al. (2017) calculated a BB contribution of 56% to OC in PM_2.5_ during winter in Mestre, while in Rome, biomass burning made up between 38 and 50% of OC in PM_10_ during the heating period (Perrino et al. 2019). In the present study, OC from BB contributed 6% and 3%, respectively, to PM_1_ and PM_10_ mean concentrations, whereas Pio et al. (2020) reported average contributions of 25% to PM_2.5_ and 19% to PM_10_ in Porto (Portugal). These outcomes point to a limited impact of BB emissions on the levels of OC and PM_10_ in our study area. In fact, PM_10_ and levoglucosan concentrations showed a different temporal behavior (Fig. 3). The most likely reasons are (1) the use of biomass as a heating source is not common in the urban area, as commented above, and (2) winters are characterized by mild temperatures and residential heating is greatly reduced during this season. It is important to bear in mind that the average winter temperature during the study period was almost 14 °C.

The ratios between levoglucosan and its isomers (Table 5) were the same as those calculated for the residential area located close to the urban sampling site (Galindo et al. 2021) and point to the use of softwood as fuel. Previous works performed in the Iberian Peninsula have also reported the predominant use of softwood for house heating (van Drooge et al. 2022; Pio et al. 2022).

Similarly to the use of levoglucosan as a tracer to estimate the contribution from biomass burning, mannitol has been used in previous works to quantitatively evaluate the contribution of fungal spores to OC and PM_10_ concentrations (Gonçalves et al. 2021; Mendes Emygdio et al. 2018). The conversion factors proposed by Bauer et al. (2002, 2008) were used. These factors were 1.7 pg mannitol/spore, 33 pg fresh mass PM_10_/spore, and 13 pg C/spore. The average contribution of fungal spores to PM_10_ and OC concentrations in Elche was 1.2 and 0.8%, respectively, with minimum values in winter (0.9 and 0.5%, respectively) and maximum in spring (1.4 and 0.9%, respectively). These values are much lower than those found at other urban areas (Gonçalves et al. 2021; Mendes Emygdio et al. 2018), which suggests that fungal spores are significantly less abundant at our sampling site. In spite of this, it is important to mention that these estimates should be considered with caution since climate differences and differences in fungal species between sites may lead to different mannitol concentrations. Therefore, there are inherent uncertainties associated with the use of this approach.

## Conclusions

The average concentrations of biomass burning tracers (levoglucosan, mannosan, and galactosan) in PM_1_ and PM_10_ samples collected in the city center of Elche were low compared with those reported for other urban sites in Europe, indicating low PM emissions from this source in the study area. The reasons are that the duration of the heating period is very low (due to mild winter temperatures) and that wood is not commonly used as fuel for house heating in the city. Although sugar alcohols are thought to come mainly from biological sources, the seasonal variation of inositol, xylitol, and sorbitol at the sampling site suggests a significant contribution of biomass burning emissions. The mass size distribution of these polyols, with a larger proportion in the fine mode, and the significant correlation coefficients with levoglucosan support this hypothesis. On the other hand, mannitol and glucose were mainly distributed in the coarse fraction, and their seasonal patterns were characterized by higher levels in spring and/or summer, which indicates that they originate primarily from biogenic emissions. Levoglucosan and mannitol were used as tracers to quantitatively estimate the contribution from biomass burning and fungal spores to OC and PM concentrations. Fungal spores contributed 1.2% to PM_10_ and 0.8% to OC. Alternatively, the average contribution of OC from biomass burning to PM_1_ and PM_10_ was 6% and 3%, respectively. These results suggest a limited contribution of biomass burning emissions to PM levels in the study area, which implies that, although all sources have to be considered in order to reduce PM pollution in the city, mitigation measures should prioritize other anthropogenic sources with a greater impact on aerosol concentrations. On the other hand, the findings of the present study suggest that sugar alcohols other than mannitol come primarily from biomass burning, indicating they are not suitable markers of biogenic sources, at least in the study area. Further research using receptor models such as Positive Matrix Factorization is needed in order to confirm these results.

## Data Availability

The datasets generated and analyzed during the current study are available from the corresponding author on reasonable request.
